# Simultaneous Determination of Cr, As, Se, and Other Trace Metal Elements in Seawater by ICP-MS with Hybrid Simultaneous Preconcentration Combining Iron Hydroxide Coprecipitation and Solid Phase Extraction Using Chelating Resin

**DOI:** 10.1155/2018/9457095

**Published:** 2018-11-13

**Authors:** Akihide Itoh, Masato Ono, Kota Suzuki, Takumi Yasuda, Kazuhiko Nakano, Kimika Kaneshima, Kazuho Inaba

**Affiliations:** ^1^Department of Environmental Science, School of Life and Environmental Science, Azabu University, 1-17-71, Fuchinobe, Chuo-ku, Sagamihara-shi, Kanagawa 252-5201, Japan; ^2^GL Science Inc., Shinjuku Square Tower 30F, 6-22-1 Nishi Shinjuku, Shinjuku-ku, Tokyo 163-1130, Japan

## Abstract

In the present study, ICP-MS with a new hybrid simultaneous preconcentration combining solid phase extraction using chelating resin and iron hydroxide coprecipitation in one batch at a single pH adjustment (pH 6.0) were developed for multielement determination of trace metal ions in seawater. In multielement determination, the present method makes it possible to determine Cr(III), As(V), Se (IV), and other 14 trace metal elements (Ti, V, Co, Ni, Cu, Zn, Zr, Ge, Cd, Sb, Sn, W, Pb, and U) in seawater. Moreover, for speciation analyses of Cr, As, and Se, the pH dependence on recovery for the different chemical forms of Cr, As, and Se was investigated. In speciation analyses, Cr, As, and Se were determined as the total of Cr (III) and a part of Cr (VI), total of As (III) and As (V), and Se(IV), respectively. Determination of total of Se and Cr(VI) remains as future task to improve. Nevertheless, the present method would have possibility to develop as the analytical method to determine comprehensively most metal elements in all standard and guideline values in quality standard in environmental water in Japan, that is, most toxic metal elements in environmental water.

## 1. Introduction

ICP-MS has excellent analytical features such as simultaneous multielement capability, extremely high sensitivity, and wide linear dynamic range for most metal elements [1–3]. So, ICP-MS makes it possible to determine comprehensively almost all heavy metals, whose standard values or guideline values were established in water quality standards for human health relating to water pollution in Japan, without any special preconcentration. However, the measurement of trace metals such as heavy metals in seawater is difficult even using ICP-MS, because the salt contents in seawater are approximately 3.5% and they cause not only matrix effect and spectral interference but also the clogging of the torch top and the orifice of cone in ICP-MS [3]. These days, a high matrix introduction (HMI) unit permits the direct introduction of seawater into ICP-MS. In addition, novel ICP-MS with tandem quadrupole mass spectrometer (QMS/QMS) as well as with an octapole reaction cell (ORC) have become commercially available, which provides efficient removal of spectral interferences due to oxide species [4]. However, ICP-MS with preconcentration and desalting remains the most efficient method for the simultaneous and sensitive determination of trace metal elements in seawater without spectral interference and matrix effect.

The chelating resin preconcentration method has excellent analytical features of nonselective multielement determination for many trace elements in seawater, along with efficient removal of matrix elements such as Na, K, Ca, and Mg [5–10]. However, it is found that the chelating resin provided poor recoveries for some oxoanion-forming elements, such as As, Se, and Cr, which are toxic and important in environmental sciences. Coprecipitation methods [11] such as lanthanum hydroxide [12–14], iron hydroxide [15–17], yttrium hydroxide [18], and magnesium hydroxide [19] are also effective as other preconcentration methods to complement chelating resin technique, because both oxoanion-forming elements and cation-forming trace elements can be concentrated using this method. However, the coprecipitation methods do not allow one to analyze toxic metal elements comprehensively, although they provided good recoveries for some oxoanion-forming elements and/or a part of transition metals. In addition, coprecipitation carrier results in high concentration of matrix components. Accordingly, performing both chelating resin preconcentration and coprecipitation complementally under control of matrix components is effective to determine many trace metal elements including toxic ones simultaneously. Yabutani et al. developed the tandem preconcentration method based on chelating resin adsorption and lanthanum hydroxide coprecipitation method that was continuously used to determine the oxoanion-forming elements and other trace elements [20]. However, this tandem method was time-consuming, because a series of preconcentration procedures including pH adjustment need to be performed for the coprecipitation after the chelating resin preconcentration. In contrast, a proposed new hybrid simultaneous preconcentration method was examined as a batch method that uses a single pH adjustment to achieve solid phase extraction using chelating resin and iron hydroxide coprecipitation. Thus, the potentials of ICP-MS with the present hybrid preconcentration method were investigated for simultaneous preconcentration and determination of the oxoanion-forming elements such as Cr, As, Se, and other trace metal elements, whose standard and guideline values were established in environmental quality standards for water pollution in Japan, even in seawater containing high concentrations of salts.

## 2. Materials and Methods

### 2.1. Instruments

An ICP-MS instrument (Agilent 7700x, Agilent Technologies Co., Tokyo, Japan), equipped with a quadrupole mass spectrometer and an octapole reaction cell (ORC), was used for the determination of trace metals in preconcentration solution of seawater. The operating conditions for the ICP-MS instrument were summarized in Table 1. In the ICP-MS measurement, the internal standard correction was performed using Be, In, and Tl as internal standard elements to correct matrix effects due to major elements [21]. The purified water (18.2 MQ cm) used throughout the present experiment was prepared by a Milli Q SP-TOC system (Nihon Millipore Kogyo, Tokyo, Japan).

### 2.2. Chemicals

The standard solutions for making the calibration curves in the ICP-MS measurements were prepared by diluting commercial multielement standard stock solutions (XSTC-622, 35 elements, 10 mg L^−1^ each), which were purchased from SPEX (Metuchen, NJ, USA). As, Cr, and Se were involved as As(V), Cr(III), and Se(IV) in XSTC-622, respectively. Nitric acid, hydrochloric acid, acetic acid, and aqueous ammonia solution were of electronics industry grade (Kanto Chemical Co., Tokyo, Japan). A single-element standard stock solution of Fe 10000 mg L^−1^ for general tests in the Japanese pharmacopoeia (Wako Pure Chemical Industries Inc., Osaka, Japan) was used as the iron solution for iron hydroxide coprecipitation.

The added standard solutions to investigate the recovery values were prepared as follows. The standard stock solutions for Cr(III), Cr(VI), and As(V) were prepared by diluting chromium(III) standard for ICP, chromium(VI) standard for ICP, and Arsenic (V) standard solution (1000 mg L^−1^ each, Merck, Darmstadt, Germany), respectively. The standard stock solutions for As(III) and Se(IV) were prepared by diluting standard solution of arsenic (III) and selenium (IV) for chemical analysis (1000 mg L^−1^ each, Kanto Chemical Co.), respectively. The standard solution for Se(VI) was prepared by extra grade of dissolving sodium selenite (Wako Pure Chemical Industries Inc.) in ultrapure water.

The chelating resin particles (InertSep ME2, 60-70 *μ*m in diameter, GL Science Inc., Tokyo, Japan) have iminodiacetic acid (p*K*_a_ = 2.98) and dimethylamino (p*K*_a_ =10.77) groups on methacrylate resin. This resin was beforehand conditioned with ethanol, 2 M HNO_3_, purified water, and 0.1 M ammonium acetate solution, which was used for chelating resin preconcentration of seawater samples. The ammonium acetate solution (pH 6) used for the pH adjustment was prepared by mixing equivalent molar amounts of acetic acid and ammonia solution.

The artificial seawater was prepared as follows using some reagents of extra grade purchased from Wako Pure Chemical Industries Inc.: 28.5 g of sodium chloride, 6.82 g of magnesium sulfate heptahydrate, 5.16 g of magnesium chloride hexahydrate, 1.47 g of calcium chloride dehydrate, 0.725g of potassium chloride, 0.084 g of sodium bromide, and 0.0273 g of boric acid were dissolved in ultrapure water. Then, the volume of the solution was adjusted to be 1 L with ultrapure water.

### 2.3. Procedure of the Hybrid Preconcentration Combing Iron Hydroxide Coprecipitation and Solid Phase Extraction Using Chelating Resin

In the preconcentration procedure, 50 mL of a sample solution was initially taken in a 50 mL plastic bottle (DigeTUBEs, SCP SCIENCE, Canada, Montreal) and then 250 mg of the chelating resin particles, 50 *μ*L of 10000 mg L^−1^ iron standard solution, 1 mL of 1.0 M ammonium acetate (buffer solution), and 100 *μ*L of 400 *μ*g L^−1^ methyl red solution (pH indicator) were added into the sample solution. The pH of the sample solution was adjusted to 6.0±0.1 using 6 M ammonia solution. As described in Results and Discussion, the optimal pH value for seawater samples in the iron hydroxide precipitation was pH 6.0, which was also appropriate for solid phase extraction using chelating resin. In the process of hybrid preconcentration procedure, it is considered that some cation-forming trace metals were complexed on the surface of the chelating resin particles and some oxoanion-forming elements such as Cr, As, and Se were adsorbed on and/or occluded in iron hydroxides coprecipitation. Changes in color were monitored during the pH adjustment for a preliminary estimation, but the final pH of the sample solution was confirmed using with a compact pH meter (Twin pH meter, Horiba Ltd., Kyoto, Japan). Next, the iron hydroxide coprecipitations formed were ripened for 2 h at 70°C in a water bath. Then, all the solutions were filtered using *ϕ*25 mm the glass fiber filter (Digi Filter, SCP SCIENCE, Canada, Montreal) with pore size of 1 *μ*m, and the chelating resin particles and iron hydroxide coprecipitates were collected on the filter simultaneously. They were then washed with 50 mL purified water several times. Trace metals from the chelating resin particles and iron hydroxide coprecipitates were eluded with an acid solution. In the elution process, 5 mL of 2.6 M nitric acid solutions was added divisionally twice (2 and 3 mL) into chelating resin particles and iron hydroxide coprecipitates on the filter. When the first 2 mL of 2.6 M nitric acid solution was added, the solution was maintained for 10 min before removing to dissolve adequately the iron hydroxide coprecipitates. Subsequently, 5 mL of ultrapure water and 3 mL of the 2.6 M nitric acid solution were added as elutants and aspirated by vacuum pump (MDA-05, ULVAC, Chigasaki, Japan). Totally, 10 mL of elutant was obtained per each sample. Hence, ca. 5-fold preconcentration was achieved for the analysis solution in the present procedure, which was subjected to the simultaneous multielement determination of Cr, As, and Se and other trace metals using ICP-MS.

In the recovery test for investigation for the added amount of Fe^3+^ and analysis of coastal seawater, 50 *μ*L of a mixed standard solution (XSTC-622, SPEX) containing 35 trace metals (1.0 mg L^−1^ each), in which Cr, As, and Se were contained as As(V), Cr(III), and Se(IV), was added into 50 mL of an artificial seawater. Then, the preconcentration procedure described above was carried out for the spiked and the unspiked artificial seawaters to estimate the recovery values. The recovery values for analyte elements were obtained as the percentages of the differences between the amounts of analyte elements in the spiked and unspiked artificial seawater samples, which was measured by ICP-MS after the present hybrid simultaneous preconcentration procedure, to the amounts added to the artificial seawater (50 ng each). In recovery test for speciation analysis of Cr, As, and Se, two kinds of recovery tests were separately performed using the stock solution for lower oxidation state of Cr(III), As(III), and Se(IV) and that for higher oxidation state of Cr(VI), As(V), and Se(VI) to avoid the oxidation-reduction reaction among these elements. 50 *μ*L of the mixed standard stock solution of Cr(III), As(III), and Se(IV) (1.0 mg L^−1^ each) was added to the artificial seawater sample to prepare 50 mL of the spiked test solution for the lower oxidation state, and those of Cr(VI), As(V), and Se(VI) (1.0 ngL^−1^ each) were added to another sample of the artificial seawater to prepare 50 mL of the spiked test solution for the higher oxidation state. These two kinds of the spiked test solutions and unspiked artificial seawater without addition of any standard solution were analyzed by ICP-MS with the present hybrid simultaneous preconcentration to investigate the pH dependence on recovery of each oxidation state of Cr, As, and Se. The recovery for each chemical form of Cr, As, and Se was calculated in a similar manner to the recovery test for investigation for the added amount of Fe^3+^ and analysis of coastal seawater described above.

### 2.4. Seawater Samples

Seawater samples were collected at the Senzu coast in Izu-Oshima Island, Tokyo, Japan. Collected samples were filtered through the membrane filters of *ϕ*47 mm with a pore size 0.45 *μ*m (Omnipore filter, Millipore, Bedford, MA, USA) immediately after sampling. The dissolved samples filtered with the membrane filters were acidified to pH 1 by adding concentrated HNO_3_ (EL grade, Kanto Chemical Co.) and then subjected to hybrid preconcentration.

## 3. Results and Discussion

### 3.1. Investigation for Added Amounts of Fe^*3*+^

**Figure 1:**
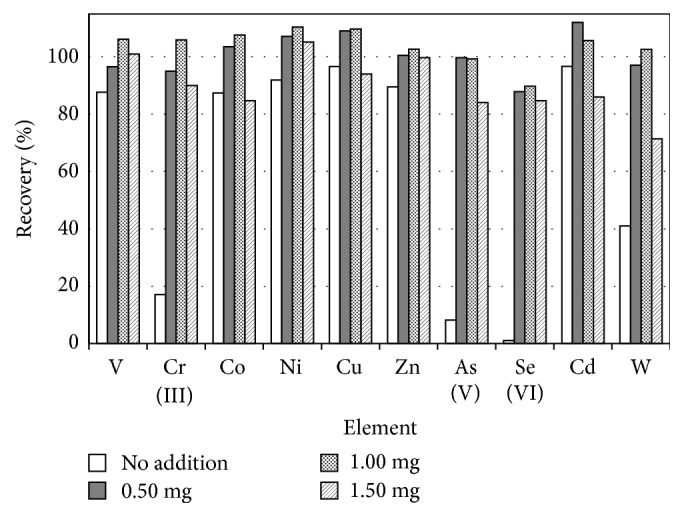
Comparison of recoveries of V, Cr(III), Co, Ni, Cu, Zn, As(V), Se(IV), Cd, and W in changing the added amount of Fe^3+^ in the hybrid preconcentration.

In the present study, iron hydroxide coprecipitation was employed along with solid phase extraction using chelating resin to develop a hybrid simultaneous preconcentration method, because Fe(OH)_3_ precipitates have a positive charge at pH 4-8 [22]. Moreover, Fe(OH)_3_ precipitates can form at pH 5-6 [16]: this acidic condition is also optimal for solid phase extraction using chelating resin. In iron hydroxide coprecipitation, the added amounts of Fe^3+^ are generally 1.5-50 mg for 50 mL of each seawater sample [15, 16]. In the present study, however, it was set as 0.5 mg for 50 mL samples, because the added amount of Fe^3+^ as coprecipitation carrier should be kept minimal to decrease total matrix concentration and not to block the performance of chelating resin particles. Thus, the optimal added amount of Fe^3+^ was investigated in the present hybrid simultaneous preconcentration. When 0 mg, 0.50 mg, 1.0 mg, and 1.5 mg of Fe^3+^ were added to 50 mL of seawater sample with 250 mg of powdered chelating resin particles, respectively, and adjusted to pH 6.0, the recoveries of V, Cr(III), Co, Ni, Cu, Zn, As(V), Se(IV), Cd, and W are shown in Figure 1. As can be seen in Figure 1, the recoveries of most analyte elements were found to be higher at 0.50 mg and 1.0 mg. From this result, it was determined that the optimal amount of added Fe^3+^ was 0.50 mg, which resulted in the smaller total matrix and provided the smaller blank values. Then, Fe concentration was maintained below 50 mg L^−1^ in the 5-fold concentrated solutions and total residual concentrations of major ions such as Mg^2+^, Ca^2+^, Na^+^, and K^+^ were below 20 mg L^−1^. Thus, the matrix effect caused by the added Fe and the residual concentration of major ions was so small to be corrected using the internal standard method [21].

### 3.2. Comparison of Recoveries in the Hybrid Preconcentration with Those in Solid Phase Extraction Using Chelating Resin

**Figure 2:**
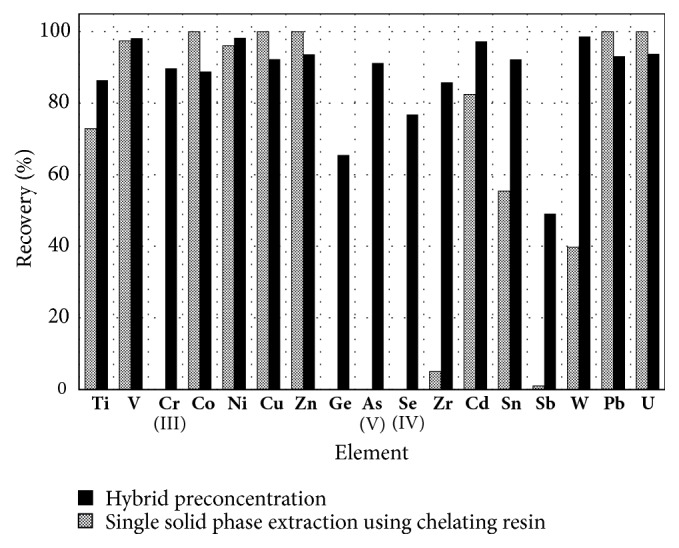
Comparison of the recoveries of Cr, As, Se, and other trace metal elements by the hybrid preconcentration with those by single preconcentration using chelating resin.

The recovery of each element in the developed hybrid preconcentration was investigated. The results are shown in Figure 2 with those in a single solid phase extraction using chelating resin. As seen in Figure 2, for single solid phase extraction using chelating resin, the recoveries of Cr(III), Ge, As(V), Se(IV), Zr, and Sb were very low below 10%, and those of Sn and W were below 60%, whereas those of V, Co, Ni, Cu, Zn, Cd, Pb, and U were over 80% and high enough for determination of trace metals in seawater. As reported in previous studies [14], it is considered that oxoanion-forming elements such as Cr, Ge, As, Se, Sb, and W provided poor recoveries in single chelating resin preconcentration, which may be ascribed to their low adsorption on the chelating resin with cation-exchange functional groups.

However, in the present hybrid preconcentration, the recoveries of oxoanion-forming elements such as As(V), Cr(III), Se(IV), Ge, Sb, and W were remarkably higher than those in single solid phase extraction using chelating resin, as seen in Figure 2. Particularly, the recoveries of As(III), Cr(III), Se(IV), and W were over 80%. Moreover, those of Ti, Cd, Zr, and Sn were 70-95% and become remarkably higher than those in single chelating resin preconcentration. The precisions of the recoveries were almost below 5% for the present hybrid method. Recovery values and precisions obtained were high enough to determine simultaneously the oxoanion-forming elements and other trace metals.

### 3.3. pH Dependence on the Recovery of Different Chemical Forms of Cr, As, and Se for Speciation Analysis

**Figure 3:**
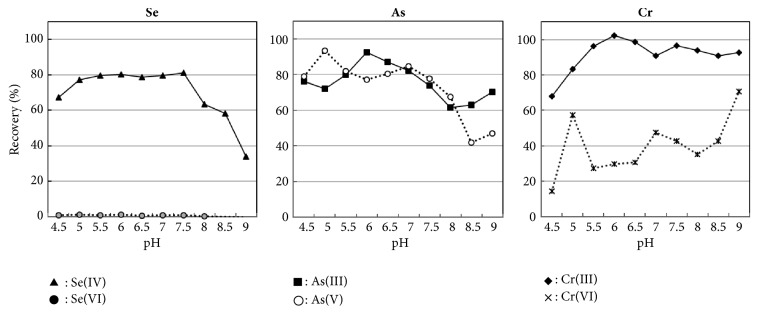
pH dependence on recovery of different chemical forms of Cr, As, and Se.

Cr, As, and Se are present as two different oxidation states in seawater and environmental water. Cr exists as either Cr^3+^ or CrO_4_^2−^ (VI) in seawater, As as either AsO_3_^3−^ (III) or AsO_4_^3−^ (V), and Se as either SeO_3_^2−^ (IV) or SeO_4_^2−^ (VI) [23]. Because the toxicity and bioavailability of these elements to aquatic animals and plants depend on the oxidation state, speciation analysis for these elements was very important to evaluate the effects on the aquatic ecosystem. Thus, the pH dependence on recovery of each oxidation state of Cr, As, and Se in seawater samples was investigated for speciation analysis. The results are shown in Figure 3. It can be seen from Figure 3 that the optimal pH to recover Se (IV), As (III), As(V), and Cr(III) simultaneously was pH 6.0. At this pH, the recovery of As was over 80%, regardless of the oxidation state. On the other hand, the recoveries of Cr(VI) and Se(VI) were ca.30% and 2%, respectively, whereas those of Cr(III) and Se (IV) were 100% and 80%, respectively. Moreover, even in different pH value, the recovery of each chemical form was not enough for its determination. Therefore, the optimal pH is also 6.0 for the speciation analyses that allows determining separately each chemical form of Cr, As, and Se. Under these conditions, the total concentrations of As, the sum of As(III) and As (V), and the concentration of Se(IV) were determined. However, it was difficult to determine separately Cr(III) and Cr(IV).

### 3.4. Simultaneous Determination of Cr, As, and Se and Other Trace Metals in Coastal Seawater

Analytical results for the oxoanion-forming elements and other trace metals dissolved in coastal seawater collected at Izu-Oshima Island are shown in Table 2 with the recovery values, blank values, instrumental detection limits (DL_instru_), and analytical detection limits (DL_anal_). The recovery values were calculated as the percentages of analyte element amounts recovered after preconcentration to those added before preconcentration (50 ng each), as described in “Experimental” section. The recovery of As in Table 2 was obtained for As (V). However, as the recovery of As(III) was as high as that of As(V) at pH 6.0 in Figure 3, the analytical result of As in coastal seawater is shown as As (III+V) in Table 2. The DL_instru_ of the analyte elements was obtained at the concentrations corresponding to 3-fold the standard deviation (3*σ*) of the background signal intensities for the blank solution (2 M HNO_3_), where the standard deviation (*σ*) was calculated from 10-times repeated measurement at each mass number. As it is confirmed that the linearities of the calibration curves for all analyte elements ranged from DL_inst_ to over 100 ng L^−1^, all analyte elements in the concentrated sample solutions were measured within the liner range of the calibration curves. The DL_anal_ was estimated from the instrumental detection limits, taking into consideration the concentration factors and recovery values. The concentrations for 17 trace metals, which were corrected by the recovery values, concentration factors, and blank values, are shown in Table 2. The observed values and relative standard deviations (RSDs) were estimated from mean values and standard deviation (*σ*) of the independent 3-times analyses. As can be seen in Table 2, the concentrations of Cr (III), As (III+V), Se (IV), and other trace metals (Ti, V, Co, Ni, Cu, Zn, Ge, Zr, Cd, Sb, Sn, W, Pb, and U) were in the range of 3.0 *μ*g L^−1^ for U to 0.012 *μ*g L^−1^ for Co, which were determined with low RSDs below ca. 10% except for Ti. The recovery values for most elements in Table 1 were large (more than 85%) enough to obtain reliable analytical data, whereas those for Ge, Se(IV), and Sb were below 70%. However, they were employed for correction of the determined values, because the RSDs for these recoveries were below 3% and the precisions were very high. The blank values for most elements were below sub *μ*g L^−1^ and low enough to correct the determined values. However, those for Co and Ge were over 10% of the observed values and relatively high. Therefore, these determined values were showed with asterisk in Table 2, as they may be less reliable than those for other elements.

## 4. Conclusion

In the present study, ICP-MS with a new hybrid simultaneous preconcentration combining the solid phase extraction using chelating resin and iron hydroxide coprecipitation in one batch at a single pH adjustment (pH 6.0) were developed for multielement determination of trace metal ions in seawater. In multielement determination, the present method made it possible to determine Cr(III), As(V), Se(IV), and 14 other trace metal elements (Ti, V, Co, Ni, Cu, Zn, Ge, Zr, Cd, Sb, Sn, W, Pb, and U). However, in speciation analyses, Cr, As, and Se were determined as the total of Cr (III) and a part of Cr (VI), total of As (III) and As (V), and Se(IV), respectively. Determination of total of Se and Cr (VI) remains as future task to improve. Nevertheless, the present method would have possibility to develop as the analytical method to determine comprehensively most metal elements in all standard and guideline values in quality standard in environmental water in Japan, that is, most toxic metal elements in environmental water.

## Figures and Tables

**Table 1:** Operating conditions for the ICP-MS instrument.

ICP-MS: Agilent 7700x	
Plasma conditions:	
RF power	1.55 kW
Plasma gas flow rate	15.0 L min^−1^ Ar
Auxiliary gas flow rate	0.90 L min^−1^ Ar
Makeup gas flow rate	0 L min^−1^ Ar
Carrier gas flow rate	1.05 L min^−1^ Ar
Sampling depth (mm from load coil)	8.0 mm
Cell gas	He mode:4.3 mL min^−1^
	H_2_ mode:6.0 mL min^−1^
Nebulizer	Micro Mist
Sample uptake rate	0.45 mL min^−1^

Data acquisition:	
Accumulation time	0.3-1.0 s / point
Data point	3 points / peak
Repetition	3 times

**Table 2:** Analytical results for Cr, As, Se and other trace metals in coastal seawater (Izu Oshima, the senzu coast) determined by ICP-MS with hybrid preconcentration combining iron hydroxide coprecipitation and solid phase extraction using chelating resin.

Element	Cell gas mode	m/z	Concentration		Recovery		Blank value/ *μ*gL^−1^	DL_instru_^(b)^/ *μ*gL^−1^	DL_anal_^(c)^/ *μ*gL^−1^
			Observed ^(a)^ / *μ*gL^−1^	RSD/%	Mean ^(a)^ / %	RSD/%			
Ti	He	47	0.027 ± 0.009	32.7	96.9 ± 0.85	0.88	n.d. ^(d)^	0.072	0.0074
V	He	51	1.87 ± 0.08	4.0	100.5 ± 0.99	0.99	0.001	0.0024	0.00024
Cr (III)	He	52	0.24 ± 0.02	7.4	96.3 ± 0.5	0.56	0.018	0.013	0.0013
Co	He	59	0.012*∗* ± 0.001	7.1	88.6 ± 0.57	0.64	0.006	0.0012	0.00014
Ni	He	60	0.22 ± 0.01	5.8	105.0 ± 3.2	3.3	0.012	0.0031	0.0031
Cu	He	63	0.17 ± 0.02	10.3	104.7 ± 1.9	2.0	0.034	0.021	0.0021
Zn	He	66	1.85 ± 0.03	1.8	86.8 ± 1.9	2.2	0.077	0.058	0.0067
Ge	He	72	0.72*∗* ± 0.04	5.2	65.5 ± 1.9	2.8	0.31	0.0018	0.00027
As (III + V)	He	75	1.34 ± 0.05	4.0	90.8 ± 0.84	0.92	0.0012	0.0024	0.00026
Se (IV)	H_2_	78	0.0324 ± 0.0002	0.8	71.1 ± 0.17	0.24	n.d. ^(d)^	0.0021	0.00029
Zr	He	90	0.055 ± 0.004	6.9	85.8 ± 2.3	2.6	n.d. ^(d)^	0.0072	0.00084
Cd	He	111	0.013 ± 0.002	11.7	91.5 ± 0.53	0.58	n.d. ^(d)^	0.0006	0.00007
Sn	He	118	0.065 ± 0.008	12.7	99.1 ± 0.57	0.57	0.0061	0.011	0.0011
Sb	He	121	0.23 ± 0.03	11.5	49.1 ± 0.39	0.80	0.0008	0.0003	0.00006
W	He	182	0.020 ± 0.001	5.1	98.6 ± 0.16	0.17	0.002	0.0029	0.00029
Pb	He	208	0.087 ± 0.01	10.4	98.9 ± 3.8	3.9	0.0023	0.0055	0.00056
U	He	238	3.0 ± 0.2	5.2	103.0 ± 0.010	0.010	n.d. ^(d)^	0.0011	0.00011

(a) Mean ±*σ*(standard deviation),n=3, The observed values with asterisk were corrected by the blank values over 10% of the observed values.

(b) DL_instru_: instrumental detection limit. (c) DL_anal_: analytical detection limit. (d) Not detected.

## Data Availability

The output data obtained to support the findings of this study are available from the corresponding author upon request.
